# Genetics of chronic respiratory disease

**DOI:** 10.1038/s41576-024-00695-0

**Published:** 2024-03-06

**Authors:** Ian Sayers, Catherine John, Jing Chen, Ian P. Hall

**Affiliations:** 1 NIHR Nottingham Biomedical Research Centre, University of Nottingham, University Park, Nottingham, UK; 2 Biodiscovery Institute, School of Medicine, University of Nottingham, University Park, Nottingham, UK; 3 University of Leicester, Leicester, UK; 4 University Hospitals of Leicester, Leicester, UK

## Abstract

Chronic respiratory diseases, such as chronic obstructive pulmonary disease (COPD), asthma and interstitial lung diseases are frequently occurring disorders with a polygenic basis that account for a large global burden of morbidity and mortality. Recent large-scale genetic epidemiology studies have identified associations between genetic variation and individual respiratory diseases and linked specific genetic variants to quantitative traits related to lung function. These associations have improved our understanding of the genetic basis and mechanisms underlying common lung diseases. Moreover, examining the overlap between genetic associations of different respiratory conditions, along with evidence for gene–environment interactions, has yielded additional biological insights into affected molecular pathways. This genetic information could inform the assessment of respiratory disease risk and contribute to stratified treatment approaches.

## Introduction

Frequently occurring chronic respiratory diseases, such as asthma, chronic obstructive pulmonary disease (COPD) and interstitial lung diseases, are the third leading cause of global deaths and accounted for 7% of global mortality in 2017 (refs. 1,2). In the UK, approximately 12 million people have a respiratory disorder, and the annual cost to the National Health Service (NHS) is around £11 billion. In the USA, costs related to respiratory conditions were estimated at around US$171 billion in 2016 (ref. 3). COPD and asthma are the commonest respiratory diseases, but interstitial lung diseases, such as idiopathic pulmonary fibrosis (IPF), are increasingly being referred to secondary care as therapeutic approaches are improving to counteract their particularly poor prognoses^4,5^.

**Fig. 1:**
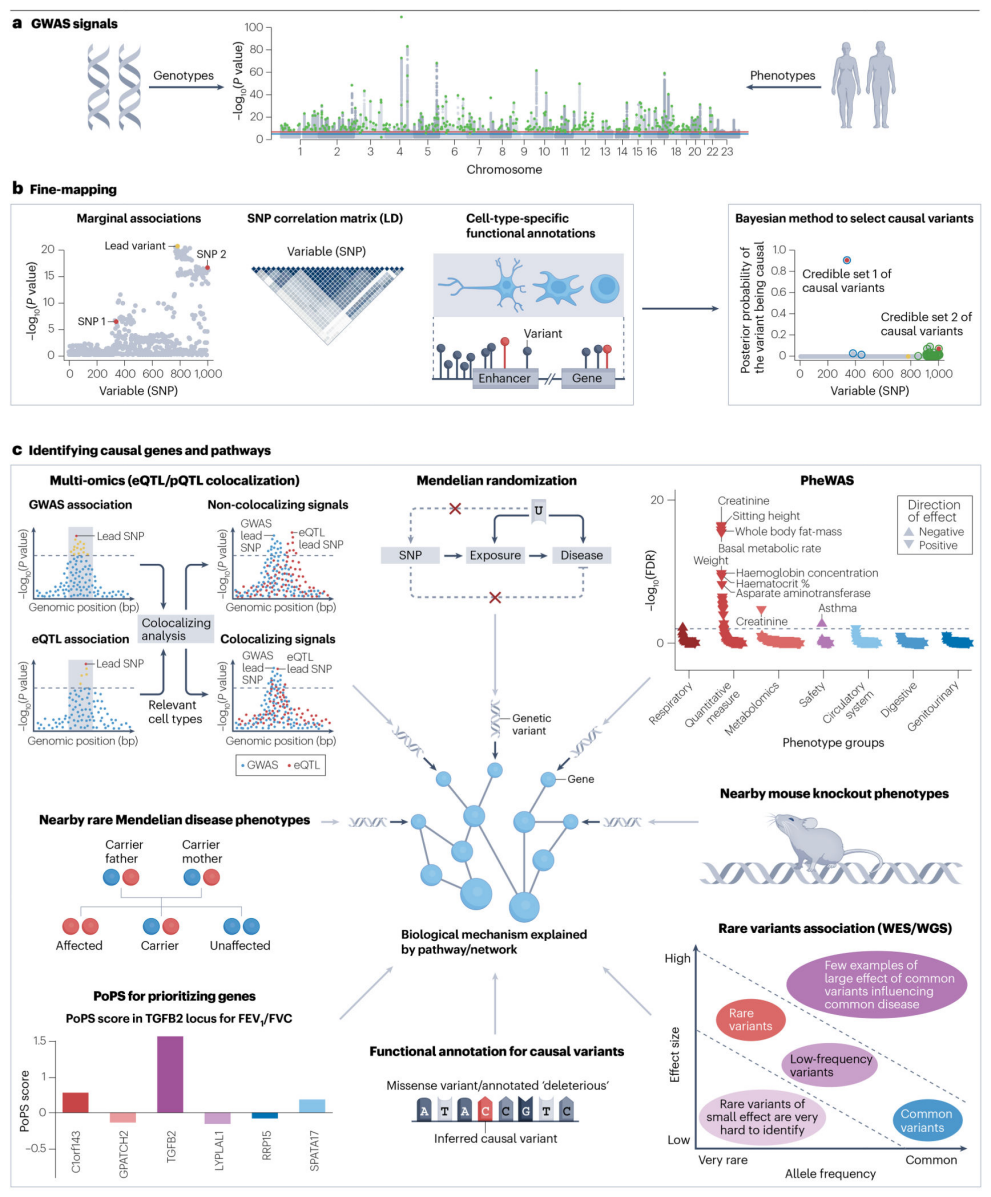
Workflow for variant-to-gene mapping. **a**, Genome-wide association studies (GWASs) are used to identify associations between genotype and respiratory phenotypes. These either use disease status as a binary trait, or quantitative traits such as the lung function variables forced expiratory volume in one second (FEV_1_), forced vital capacity (FVC), peak expiratory flow rate and the FEV_1_/FVC ratio. **b**, The genetic associations are further refined using fine-mapping approaches, including Bayesian methods^103,104^. Next, identifying the location of the causal variants can provide further insight into the mechanism — although most putative causal single-nucleotide polymorphisms (SNPs) are in non-coding regions, which require specific approaches to address function. **c**, Having identified the sentinel (lead credible) variant, the implicated gene is investigated using a range of omic and linkage disequilibrium (LD) score regression approaches^105,106^. For example, one can determine whether a variant is in a region important for transcriptional regulation by testing for enrichment of histone marks such as H3K27ac, H3K9ac, H3K4me3 or H3K4me1. Tools such as GARFIELD^107^ assess whether a variant is in or near a DNAse 1 hypersensitivity site; variants in such regions are more likely to be driving functional effects. Increasing numbers of expression and protein quantitative trait locus (eQTL and pQTL) datasets can help to identify the gene for which altered expression contributes to a given genetic association^108–110^. Other approaches include searching for nearby rare variants in whole-exome sequencing data (for example, in the UK Biobank^111,112^), prioritizing genes with a known Mendelian disease with a respiratory phenotype, and considering data from mouse knockout models to look for respiratory phenotypes with potential genes of interest. PoPS, polygenic priority score, is a gene prioritization method that leverages GWAS summary statistics and incorporates data from bulk and single-cell expression datasets, curated biological pathways and predicted protein–protein interactions^113^. Finally, biological mechanisms can be further explored using pathway approaches by grouping the genes identified as likely to be driving effects, and by exploring other phenotypes using phenome-wide association study (PheWAS) approaches^114^. U, potential confounders; WES, whole-exome sequencing; WGS, whole-genome sequencing; FDR, false discovery rate; bp, base pair. Panel **b** is partially adapted from ref. 103, CC BY 4.0 (https://creativecommons.org/licenses/by/4.0/). Panel **c** is partially adapted from ref. 115, Springer Nature Limited. Panel **c** partially adapted from ref. 116, CC BY 4.0 (https://creativecommons.org/licenses/by/4.0/).

For many years, diseases such as COPD and asthma were considered to be a result primarily of environmental factors, such as smoking or exposure to environmental pollutants. However, heritability estimates have clearly shown that genetic factors substantially contribute to the risk of developing chronic respiratory diseases^6,7^. Over the past 15 years, large-scale efforts driven by initiatives such as the Cohorts for Heart and Aging Research in Genomic Epidemiology (CHARGE), the International COPD Genetics Consortium (ICGC), the Trans-National Asthma Genetic Consortium (TAGC) and SpiroMeta have facilitated a rapid increase in our understanding of the genetic basis of common lung diseases^8–15^. Discovery efforts have been greatly aided by population-based studies such as the UK Biobank, as well as the increasing availability and size of cohort studies for specific diseases. Most efforts to identify the genetic underpinnings of chronic lung disease have used genome-wide association studies (GWASs) (Table 1). However, whole-exome and whole-genome sequencing data from cohorts with information on respiratory phenotypes are likely to provide additional value, particularly in identifying the contribution from rare variants^16^. Furthermore, new transcriptomic and proteomic datasets are helping us to understand the functional effects of causal variants underlying these associations (Fig. 1).

Currently, many medications for patients with common respiratory diseases are targeted at controlling symptoms rather than directly modifying the disease process. A better understanding of the genetic basis and affected molecular pathways of chronic lung diseases would enable the development of novel therapeutic approaches. In addition, these advances may help to identify groups at increased risk of developing respiratory disease — potentially facilitating early intervention — and subgroups of patients who are likely to respond better to specific treatments. Indeed, drug targets for diseases with supporting genetic information are more likely to become therapeutic agents^17,18^.

This Review covers the current state of knowledge on genetic associations for common respiratory disease and quantitative traits relating to lung function. We first explore how defining the genetic contribution to quantitative traits related to lung function can provide insight into common respiratory diseases. We then review insights from disease-specific GWASs for COPD, asthma and interstitial lung diseases, and discuss the extent and implications of overlap between genetic associations of these respiratory diseases, as well as the evidence for gene–environment interactions. Finally, we consider how genetic information can inform the assessment of respiratory disease risk and contribute to stratified treatment approaches. We do not cover rare respiratory diseases, pulmonary vascular disease or diseases for which the genetic contribution is predominantly monogenic (such as cystic fibrosis), or the genetic basis for various lung cancers (see refs. 19–21 for recent studies covering these subjects).

## Quantitative genetics of lung function traits

The use of quantitative traits for GWASs, compared with studies assessing binary traits, increases the statistical power in identifying genetic associations. Possible genetic associations with lung function traits, and/or disease risk, were primarily investigated through candidate gene studies before 2010; however, these findings have generally not been validated using GWAS approaches (for example, see refs. 8,22).

### Measuring lung function with spirometry

**Fig. 2:**
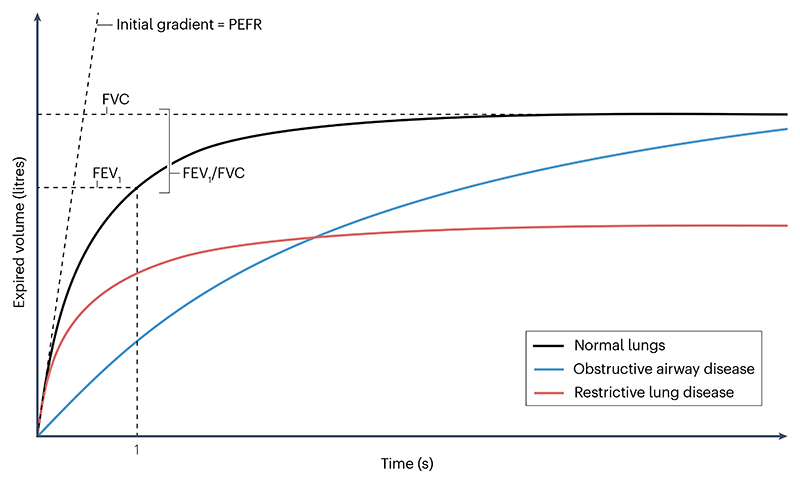
Spirometry and lung function traits. Spirometry provides quantitative lung function traits by measuring the expired volume of air over time. Deviations from healthy lungs (black line) caused by either obstructive airway disease (blue line) or restrictive lung disease (red line) are captured by key metrics calculated from the resulting curve: these include the forced expiratory volume in one second (FEV_1_), which is the volume of air expelled from the lungs in one second in a forced expiratory manoeuvre; the forced vital capacity (FVC), which is the total volume of air expelled from the lungs during a forced expiratory manoeuvre; the FEV_1_/FVC ratio; and the peak expiratory flow rate (PEFR), which is the maximum flow rate recorded during a forced expiratory manoeuvre.

Lung function is primarily tested using spirometry, which measures the total amount of air exhaled in a forced respiratory manoeuvre, termed forced vital capacity (FVC), and the amount of air exhaled during the first second of the manoeuvre, termed the forced expiratory volume in one second (FEV_1_). One can quantify other related parameters, such as the FEV_1_/FVC ratio and the peak expiratory flow rate (PEFR), which can be also used as quantitative traits (Fig. 2). Relative to healthy lungs, a reduced FEV_1_/FVC ratio indicates airflow obstruction — a key feature of COPD and some other respiratory diseases. A reduced FVC, with a preserved FEV_1_/FVC ratio, indicates lung restriction, either because of reduced chest wall motion or parenchymal lung disease reducing the ability of the lung to expand fully. This phenomenon is seen in interstitial lung diseases, of which IPF is the most common in clinical practice. Last, a reduced PEFR — usually measured using a peak-flow meter — indicates airflow limitation, and hence generally aligns with FEV_1_. Other lung function parameters, such as gas transfer, are more time-consuming to measure, and are therefore not usually assessed in large population studies, resulting in fewer data available for quantitative trait analyses.

### Insights from genome-wide association studies

**Fig. 3:**
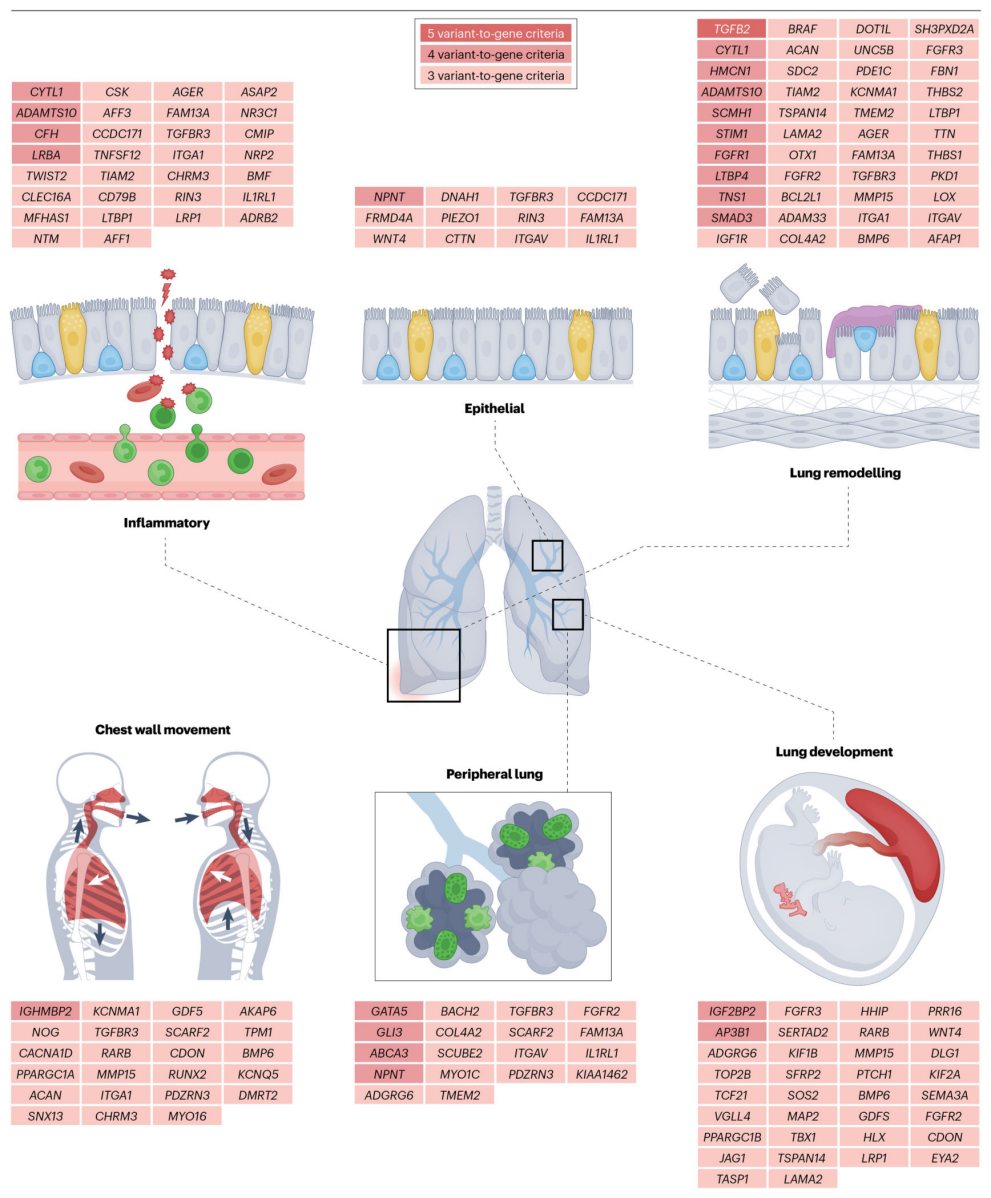
Key genes and pathways implicated by GWASs of lung function. Implicated genes are grouped based on organ function. The colour of the gene box indicates the number of criteria met that support variant-to-gene mapping using the approach shown in Fig. 1 and described in detail in ref. 19. Reprinted from ref. 22, CC BY 4.0 (https://creativecommons.org/licenses/by/4.0/).

The first meta-analyses of GWASs for lung function were performed by the SpiroMeta and CHARGE consortia and published in 2010 (refs. 11,23). These initial efforts were followed by several studies of increasing sample size^8,10,13^. The most recent^22^ meta-analysis involved 580,869 participants and identified 1,020 independent genetic associations for different lung function traits: the FEV_1_, the FVC, the FEV_1_/FVC ratio and the PEFR. These genetic associations implicated 559 genes from a systematic variant-to-gene mapping framework in which variants identified by GWASs were further refined using additional data (Fig. 1). Interestingly, the 1,020 genetic associations were seen to be enriched in non-coding regions important for the regulation of gene expression in cells relevant to lung disease, including alveolar type 1 cells, myofibroblasts, bronchial epithelial cells and in whole adult and fetal lungs. To gain further insight into the mechanisms, this study used ConsensusPath DB^24^ to test whether the 559 implicated genes were enriched in particular biological pathways and tissues (Fig. 3). Some of the pathways found had previously been implicated in lung function (for example, elastic fibre formation) but others were directly implicated here for the first time (for example, PI3–Akt signalling). Notably, many of these genetic associations were also seen in cohorts of children and young adults, in addition to older people, suggesting that many effects may be driven by the regulation of pathways important in lung growth. This observation is consistent with current epidemiological evidence showing that a low FEV_1_ in early adulthood is an important contributor to COPD risk^25^. Overall, the NHGRI–EBI GWAS Catalog currently lists 3,691 genetic associations for FEV_1_/FVC to date (although not all associations are independent), emphasizing the substantial role of genetics in determining lung function.

GWAS approaches have also helped to define effect sizes of genetic variants contributing to lung function. Although many common genetic loci contribute to variability in lung function, the effect size of any single genetic variant is small. For example, the 1,020 genetic associations identified in the most recent meta-analysis described above^19^ together explain 33.0% of FEV_1_/FVC heritability (21.3% for FEV_1_, 17.3% for FVC and 21.4% for PEF; see also Table 1).

### Insights from phenome-wide association studies

Phenome-wide association studies (PheWASs), which explore the association between a given genetic variant and multiple phenotypes, provide further insight into the contribution of genetic variants to specific mechanisms underlying lung function. Notably, PheWASs can be explored with individual or combinations of SNPs. PheWASs for genetic variants associated with lung function traits^19^ have identified, as expected, strong associations with other respiratory phenotypes — in particular, traits relevant for COPD — but also many other phenotypes. Further analyses can be done by generating a polygenic risk score (PRS, also known as a genetic risk score if used in targeted studies when considering a smaller number of genetic variants or specific known variants strongly associated with a trait of interest; here, we use the term PRS for consistency). The same study generated a PRS using pathway-partitioned SNPs to demonstrate that lung function variants (in specific pathways) are associated with a range of other phenotypes. For example, the PRS for a lower FEV_1_/FVC ratio specific to the elastic-fibre-formation pathway was associated with an increased risk of inguinal, abdominal, diaphragmatic and femoral hernias, of diverticulosis, of arthropathies, of hallux valgus and of genital prolapse; and with a reduced risk of carpal tunnel syndrome and lower body mass index^22^.

### Limitations of lung function analyses

There are important limitations of lung-function-based analyses to consider. First, nearly all large population-based cohorts in which lung function has been measured have not undertaken bronchodilator reversibility testing. This measurement is needed to assess any reversible component of airflow obstruction. Asthma is characterized by the presence of reversible airflow obstruction, whereas irreversible airflow obstruction is a key feature of COPD. This limitation is likely to have contributed to the current absence of replicated genetic associations specifically assessing the reversible component of airflow obstruction, although one study in patients with COPD found some suggestive associations^26^. Second, most studies measure lung function at only one point in time and therefore prohibit the mapping of genetic variants affecting rate of lung growth or decline. Third, most cohorts used in GWASs lack diverse ancestries. Although there are efforts to increase the availability of data from non-European-ancestry populations, such sample sizes remain much smaller, leading to limited statistical power. Increasing the diversity of ancestries in GWASs is important for improving the fine-mapping resolution and for developing novel therapeutic interventions that are applicable across populations^27,28^.

## GWASs of respiratory diseases

In addition to studies using lung function as a quantitative trait for GWAS analyses, other studies have explored chronic respiratory diseases as binary traits.

### COPD

COPD causes breathlessness that is due to either airspace damage (emphysema) or airflow obstruction^1,2^, and is caused when susceptible individuals are exposed to certain environmental stimuli — in particular, smoking and poor air quality. The NHGRI–EBI GWAS Catalog currently (as of December 2023) lists 1,150 genetic associations (with varied levels of supporting evidence) for COPD across 113 different studies^29^. One challenge in exploring genetic associations for COPD is the trade-off between more extensive phenotyping and sample size. One comprehensive COPD genetics resource that takes into account both breadth of phenotyping and sample size is COPDGene^30^, which includes a cohort of 10,198 individuals, most of whom had computed tomography scans and longitudinal follow-up^31^.

Many genetic associations for COPD and lung function traits overlap, which is unsurprising given that an FEV_1_/FVC ratio of less than 0.7 is one of the criteria for a diagnosis of COPD. Indeed, a PRS developed for lung function is strongly predictive of COPD; a recent study using a multi-ancestry lung function PRS found a 5.2-fold higher risk of COPD in the highest-risk decile compared to the lowest-risk decile^22^. Furthermore, many of the individual genes and biological pathways implicated through GWASs of COPD overlap with those identified using the FEV_1_/FVC ratio or the FEV_1_ as phenotypes. Indeed, pathway-specific PRSs defined using genetic associations for the FEV_1_/FVC ratio show strong evidence of association with COPD phenotypes in the UK Biobank when studied using PheWAS approaches. Amongst others, the elastic fibre formation, PI3K–Akt signalling, signal transduction and hypertrophic cardiomyopathy pathways have been implicated in COPD^22^.

Accurate phenotyping for COPD is essential to capture its full genetic underpinnings. For example, self-reported COPD is less reliable as a phenotype, which probably explains why associated genetic variants have smaller effect sizes when measured using COPD as defined from electronic health records compared to COPD as defined by quality-controlled spirometry data^32^. Additionally, extensive phenotyping helps to exclude other causes of airflow obstruction (for example, asthma or bronchiectasis) and allows sub-phenotypes of COPD to be explored in more detail. For instance, imaging data is required to distinguish two pathologies in COPD: airway disease with inflammation (leading to airway narrowing and often chronic bronchitis with increased sputum production) and airspace destruction (known as emphysema). To explore this further, COPDGene have performed GWASs using computed tomography image phenotypes and identified five loci associated with emphysema-related phenotypes, two loci associated with gas-trapping phenotypes and one locus associated with airway-related phenotypes. These loci included previously known COPD genetic associations, including the *HHIP, 15q25* and *AGER* loci, as well as novel genetic associations near *SERPINA10* and *DLC1*^33^. However, many more associations are likely to exist because the sample size, and therefore detection power, was limited.

The additional insight to COPD risk variants provided by GWAS approaches is well illustrated by the Z variant (*rs28929474* T) in *SERPINA1*. Observations dating back 70 years noted that individuals homozygous for the Z allele are at increased risk of COPD owing to α1-antitrypsin deficiency, which causes emphysema. However, despite previous reports suggesting that heterozygous individuals for the Z allele may also be at increased risk of COPD^33,34^, standard GWAS approaches did not identify *SERPINA1* as a risk gene for quantitative measures of lung function or COPD. More recent PheWASs have helped to explain this discrepancy by showing that heterozygous individuals are taller, and therefore have higher lung function values^35^, which effectively balances out the Z allele effect on lung function using the standard additive genetic models applied in nearly all GWASs^36^. This finding has potential implications for exploring the effects of other rare variants which, specifically for COPD, may account for up to half the total heritability (about 36%)^37^.

### Asthma

GWAS approaches have also enhanced our understanding of the genetic basis of asthma. Since the first GWAS on asthma in 2007 (ref. 38), more recent studies have increased both the number of variants assayed or imputed and cohort sizes (see recent reviews^39,40^). At the time of writing, the NHGRI–EBI GWAS Catalog describes 194 GWASs examining asthma as a trait, which have in total identified 3,291 genetic associations. The largest study, a recent multi-ancestry meta-analysis using data from the Global Biobank Meta-analysis Initiative (153,763 cases and 1,647,022 controls), identified 179 asthma-associated loci^41^. Interestingly, downstream analyses implicated 468 genes and ultimately suggested that the genetic architecture is largely shared across individuals of all the ancestries studied. The affected genes and pathways that have been confidently identified (which remains a challenge to do) implicate broad biological-function areas relevant to asthma, including inflammation (*IL4, IL13, IL33* and HLA genes), cell activation (*CD28*), transcription factors (*GATA3* and *STAT5)* and complement and innate receptors (*TLR1* and *TLR6)*. Airway structure and remodelling mechanisms have also been implicated, including epithelial cell homeostasis through *MUC5AC* and proteases and genes important for extracellular matrix regulation. Of interest, the genetic associations for asthma show some overlap with genetic associations for COPD (discussed in more detail below), and allergic diseases, including allergic rhinitis and atopic dermatitis, suggesting common mechanisms underlying genetic effects^42^. Furthermore, PheWAS approaches demonstrate that other conditions — digestive disorders, neuropsychiatric diseases, type 2 diabetes and rheumatoid arthritis — also show overlaps with genetic associations with asthma.

A major advance would be the ability to predict asthma outcomes from genetic information. Early studies using a PRS calculated from genetic variants for asthma were relatively poor at predicting relevant phenotypes, including early-onset disease, incompletely reversible airflow obstruction and hospitalization^43^. However, two recent studies have shown that the predictive power of a PRS for asthma — as well as for COPD — has improved as the number of identified genetic associations for asthma has increased^44,45^. In the first study, this updated asthma PRS, based on UK Biobank and Trans-National Asthma Genetic Consortium summary data, was significantly associated with asthma but not with COPD; by contrast, a PRS based on spirometry GWAS signals was significantly associated with both asthma and COPD (although there was extensive heterogeneity across the cohorts)^44^. In the second study, a PRS based on the Global Biobank Meta-analysis Initiative asthma summary statistics was moderately predictive of asthma outcomes in white British participants in the UK Biobank^45^. Last, as anticipated, a PheWAS for asthma PRS found the most strongly associated phenotypes to be asthma-related traits, including wheezing, the FEV_1_/FVC ratio, diagnosis of hay fever/allergic rhinitis or eczema and eosinophil count^45^.

The largest GWASs for asthma to date have included all available data from patients with a diagnosis of asthma. However, some studies have refined the datasets to address the heterogeneity observed in clinical presentation, particularly in terms of adult-onset versus childhood-onset asthma^41,46^, moderate to severe asthma^47,48^ and asthma exacerbations^49,50^. Overall, these studies have provided some supporting evidence for genetic associations of greater relevance to subtypes of asthma but have not generally identified many subtype-specific new genetic associations. For example, variation in the *ORMDL3* locus has been shown to be more relevant for childhood-onset asthma^41^. More recently, an alternative approach addressed asthma heterogeneity by computationally identifying 22 comorbid disease patterns and then performing GWASs to ultimately identify 109 loci, including subgroup-specific loci^51^. This approach provided additional insight into relevant molecular pathways, such as additional genes involved in airway inflammation and T cell activation, and the differentiation of a subgroup of asthma with musculoskeletal co-morbidities.

**Fig. 4:**
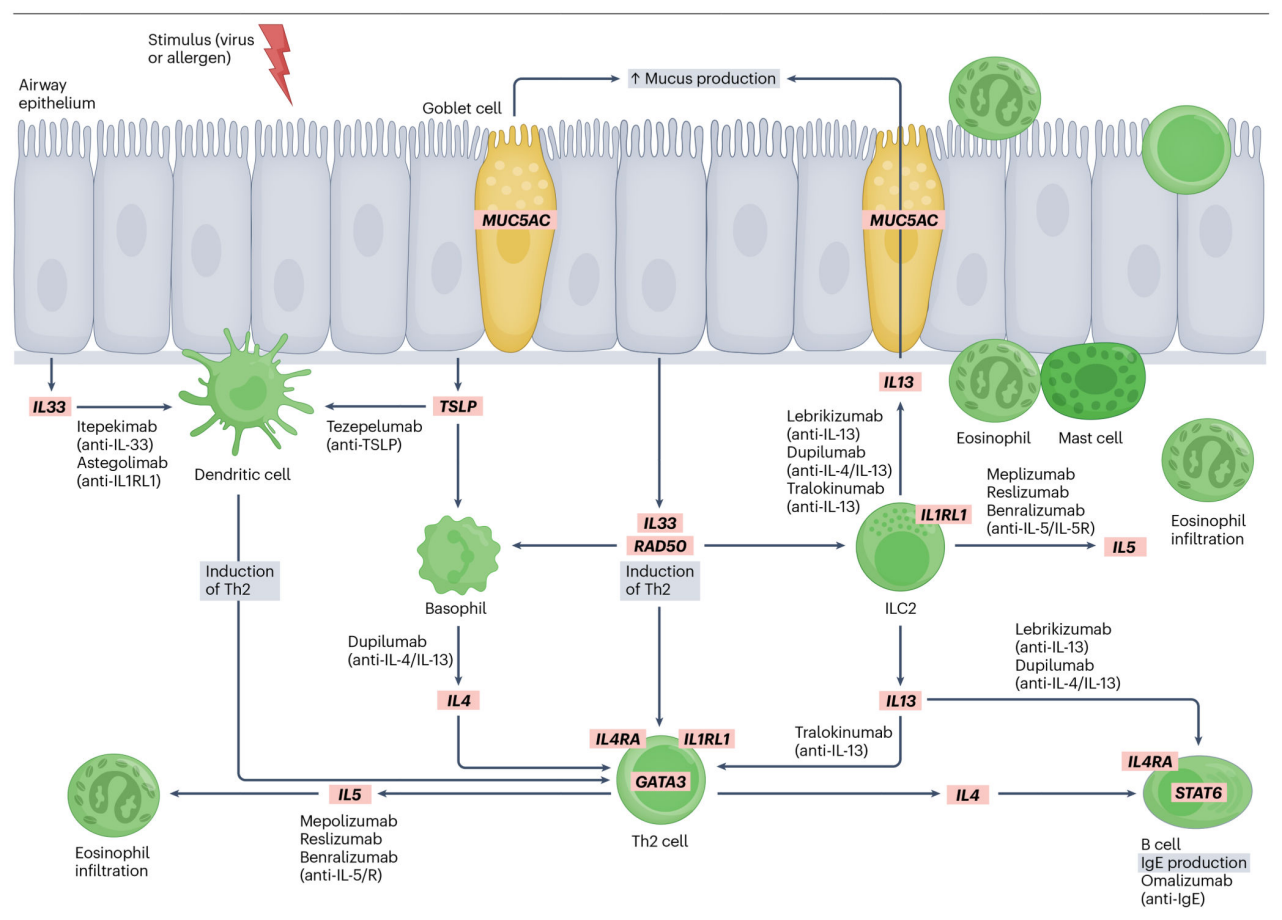
Genes and pathways identified in GWASs of asthma targeted by recently developed biological therapies. In asthma, the airway epithelium is stimulated by environmental factors such as viruses or allergens, which lead to the release of alarmins such as IL-25, IL-33 and TSLP. These alarmins then lead to an inflammatory response involving multiple cell types (for example, dendritic cells, basophils, ILC2 and Th2 cells), ultimately resulting in type 2 inflammation characterized by high levels of IL-4, IL-5 and IL-13. Candidate causal genes identified from genome-wide association studies (GWASs) are shown in red boxes and include genes encoding cytokines (for example, *IL13, IL4, IL5* and *IL33*), cytokine receptors (for example, *IL1RL1*, which is a IL-33 receptor, and *IL4RA*), signalling molecules (for example, *GATA3, RAD50* and *STAT6)* and downstream effectors (for example, *MUC5AC*, leading to mucus production). Biological therapies in development and/or in clinical practice targeting these pathways are shown, including anti-IL-13 (lebrikizumab, tralokinumab), anti-IL-4/13 (dupilumab), anti-IL-33 (itepekimab), anti-IL1RL1 (astogolimab), anti-TSLP (tezepelumab), anti-IL-5 (mepolizumab, reslizumab), anti-IL-5R (benralizumab) and anti-IgE (omalizumab). Adapted with permission from ref. 117, Elsevier.

Overall, recent work has progressed towards identifying genetic variants, genes and pathways that are related to asthma susceptibility and disease mechanisms. This research has contributed to the development of several biologic therapies coming to the clinic that target identified genes and pathways, including *IL5, IL33, IL1RL1, TSLP* and *IL4R* (Fig. 4). For example, rare-variant-based approaches highlighted IL-33 as a viable drug target by identifying a rare loss-of-function variant that alters *IL33* splicing and leads to a truncated protein that confers protection from asthma risk^52^. A key challenge in further identifying the genetic basis of asthma is the lack of large studies with clinically or biomarker-defined subgroups of disease. This type of study is necessary to reduce heterogeneity and to better identify the genetic drivers of specific asthma subgroups, enabling greater understanding and management personalized to the individual patient.

### Overlap between asthma and COPD

**Fig. 5:**
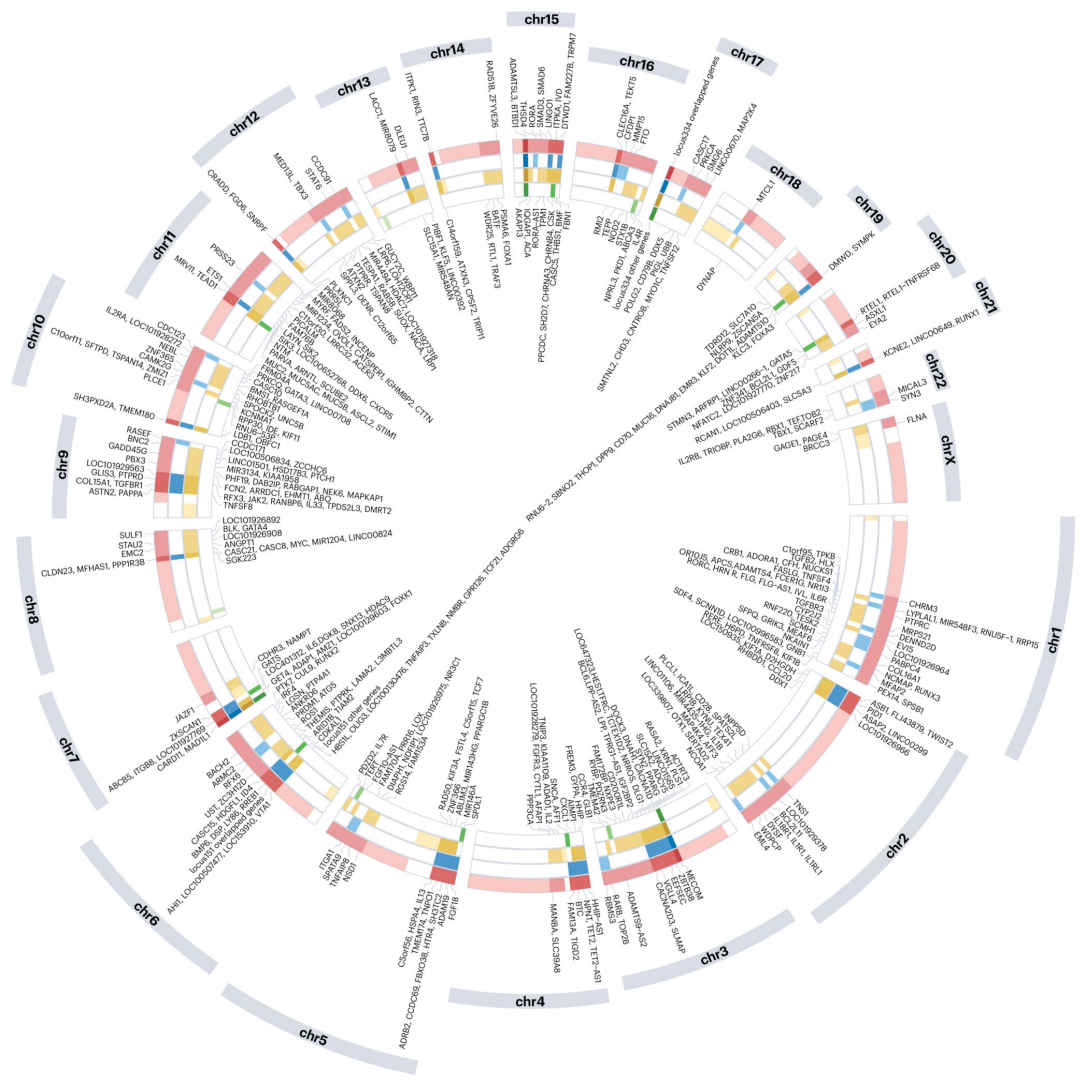
Circle plot of genetic loci implicated in one or more respiratory trait. Only regions of the genome showing association with at least one relevant phenotype are shown. Genetic associations overlapping across respiratory traits were defined using a distance-based approach. Specifically, the sentinel variants for genetic associations must be more than 2 megabase pairs apart to be considered distinct genetic loci. Lung function^22^ loci are shown in red, chronic obstructive pulmonary disease (COPD)^6^ loci in blue, asthma^19,38,40–42,46,47,49,56,59,60,118–150^ loci in orange and idiopathic pulmonary fibrosis (IPF)^151,152^ loci in green. Darker shading indicates that loci were implicated in more respiratory traits. Genes implicated by more than one trait are annotated on the outside of the plot; genes implicated for one trait are shown on the inside of the plot. There are two loci on chromosome 6 and 17 implicating a large number of genes (too many to show in the figure): locus 151 genes implicated by more than one trait (*AGER, HLA-B, HLA-DQA1, HLA-DQB1, ITPR3, MICA, SCUBE3*); locus 151 genes implicated by only one trait (*HIST1H2BD, LOC401242, HCG4B, TRIM26, IER3, MIR6891, MICB, AIF1, HLA-DRB1, HLA-DPA1, HLA-DPB1, GRM4*); locus 334 genes implicated by more than one trait (*EFCAB5, GSDMA, GSDMB, KANSL1, LOC102724596, ORMDL3, SUZ12P1, THRA*); locus 334 genes implicated by only one trait (*LRRC37B, ASIC2, SLFN5, LHX1, C17orf96, ERBB2, ZPBP2, PSMD3, SMARCE1, JUP, STAT5B, ATXN7L3, MAP3K14-AS1, MAP3K14, SPPL2C, CDC27, TBX21, ZNF652, LOC101927274*). Note that, for clarity, genomic distances are not to scale.

Asthma and COPD are distinct conditions but share several clinical and pathological features, particularly in more severe or later stages of disease. Estimates of the genetic correlation between asthma and COPD range from 0.38 to 0.50 (the strongest correlation being between COPD and moderate-to-severe asthma^53^), suggesting that there is also a substantial proportion of shared genetic aetiology^15,53,54^. However, until 2016, only variants within the HLA region — which encodes the human leukocyte antigen or major histocompatibility complex, MHC — had been shown to be associated with both asthma and either COPD or lung function in European populations. The first non-HLA signal independently associated with both asthma and lung function was near *LRP1* and *STAT6* on chromosome 6, potentially related to interleukin signalling^55,56^. Subsequently, as sample sizes have grown, an increasing number of overlapping loci have been identified, including a signal near *ORMDL3*, the first locus identified in GWASs of asthma^38^. There is now known to be substantial overlap in the genes involved in asthma and COPD or lung function (Fig. 5). These overlapping genes implicate inflammatory pathways, such as cytokines (for example, *IL1R1, IL1RL1, IL2RA, IL13* and *IL18R1*) and transcription factors (for example, *SMAD3* and *STAT6*) involved in cytokine signalling. Other overlapping genes implicate pathways for airway development or remodelling such as epithelial cell function (for example, *ORMDL3/GSDMA*), proteases (for example, *ADAM19* and *ADAMTSL3*) and many others with less clear functional roles.

Individuals with clinical features of both asthma and COPD have sometimes been referred to as having ‘asthma–COPD overlap’ syndrome (ACO). Initial GWASs of ACO did not identify any variants exceeding the genome-wide significance threshold, but these GWAS were probably limited by power^57,58^ and by inconsistent definitions of ACO. The largest GWAS of ACO to date identified eight significantly associated loci, most of which had previously been associated with asthma, COPD or lung function. One variant, an intergenic SNP on chromosome 5 (present in 1.5% of individuals assayed), was unique to ACO but requires further replication^22,41,53,59,60^.

In summary, findings to date on COPD and asthma overlap suggest a spectrum of shared genetic effects. Some variants predominantly influence asthma and are associated to a lesser extent with COPD, whereas others primarily influence COPD. To further disentangle the genetic basis of ACO, phenotypes based on biomarkers (rather than clinical or epidemiological case definitions) may help to address issues of inconsistency. Additionally, other ’omics methodologies may help to provide additional support in identifying causal genes when sample sizes are too small for GWAS approaches^61^.

### Interstitial lung diseases

GWASs of interstitial lung diseases have focused predominantly on IPF, with the first GWAS being performed in 2008 in a Japanese cohort. This study implicated *TERT*, which encodes the telomerase reverse transcriptase that catalyses the addition of telomeric repeats to the ends of chromosomes^62^ (reviewed in ref. 63). More recently, GWASs of IPF have substantially increased in size, driven by the formation of consortia and access to worldwide biobanks. In addition, *SPC* and *SFTPA2* have been implicated in interstitial lung disease pathogenesis from studies of familial interstitial lung disease^64,65^. At the time of writing, the NHGRI–EBI GWAS Catalog describes 42 GWASs that have studied interstitial lung disease as a trait, identifying 210 genetic associations. To date, the largest, multi-ancestry GWAS on IPF used 11,160 cases and 1.4 million controls to identify 25 independent IPF-associated loci^66^. This study confirmed a previous, highly reproducible genetic association at *MUC5B*, which plays a role in regulating airway mucins, but also — interestingly — showed different effect sizes based on gender. The *MUC5B* promotor variant (*rs35705950*) confers a threefold-increased IPF risk factor, which is the largest genetic risk factor known for this condition^66^. The variant is associated with increased expression of MUC5B^67,68^, although the exact mechanism by which this effect contributes to disease remains to be defined. Importantly, this variant has also been associated with survival in IPF^69^.

GWASs of interstitial lung diseases have also implicated genes related to telomere biology (for example, *TERT* and *RTEL1*), profibrotic signalling (*AKAP13* and *DSP*), mitotic spindle assembly (*KIF15* and *MAD1L1*) and mTOR signalling (for example, *DEPTOR*). However, despite these findings, our understanding of the underlying biology is limited. Other interesting findings on IPF include: greater effect sizes for genetic associations in the individuals recruited by clinic rather than biobanks; significant between-study heterogeneity; and some ancestry-specific effects^66^.

In addition to case-control studies, prior work has also sought to understand genetic associations with specific clinical features in IPF. For example, one study of 1,329 individuals performed a GWAS for longitudinal change in lung function and gas transfer and implicated an antisense RNA gene for *PKN2*, a Rho and Rac effector protein^70^. Similarly, analyses of transplant-free survival in 1,878 individuals implicated *PCSK6*, which is thought to be involved in apoptosis and cancer invasiveness^71^. More recently, a whole-genome sequencing study consisting of 2,180 cases and 2,457 controls identified several loss-of-function variants in previously identified genes, including *TERT* and *RTEL1*, using gene-based analyses. From these findings, the authors suggested that additional rare variant associations contribute to IPF risk. Their overall estimate of SNP heritability of IPF was 32%^72^. In a recent study, a PRS based on IPF summary statistics obtained from GWAS data was shown to be associated with IPF diagnosis even when excluding the established *MUC5B* signal^73^. Interestingly, this PRS was also associated with interstitial lung abnormalities and progression^73^.

Several key challenges remain to be overcome before we can fully understand the genetic basis of interstitial lung diseases. Looking forward, studies should focus on increasing and diversifying cohort sizes to identify new genetic variants of importance, better clinical- or biomarker-driven definitions of cases and translating existing associations to genes and pathways to define opportunities for early intervention.

### Overlap of genes implicated in lung function variability, asthma, COPD and IPF

Unsurprisingly, given that most lung diseases result in abnormal lung function measurements, genetic variants associated with lung function variability overlap considerably with those contributing to respiratory disease risk. For example, because a reduced FEV_1_/FVC ratio is a key diagnostic criterion for COPD, one would expect overlap between genetic variants associated with reduced FEV_1_/FVC ratio and COPD. However, such an overlap may not always be identified because there is greater power in GWAS approaches to study a quantitative (FEV_1_/FVC ratio) rather than a discrete (COPD) trait. However, taken together, there is, as expected, an overlap in the implicated genes for each of the individual respiratory diseases (Fig. 5). Examples of the overlap between variants related to lung function variables, asthma and COPD are described in the relevant section above. The locus with perhaps the most obvious overlap between genetic associations seen for multiple disease traits is the *MUC5AC* and *MUC5B* region, which suggests a key role for mucins in airway pathology. Notably, however, the individual signals observed at this locus are not the same for the different diseases, indicating different effects on mucin biology driven by genetic variation in each disease. This example illustrates the importance of ascertaining whether apparently overlapping genetic associations are driven by the same or different variants, which is usually determined through co-localization analyses.

For the overlapping genetic associations between lung function traits and respiratory disease, a key question is: does altered lung function contribute to disease risk or does disease cause altered lung function? For example, a genetic variant might affect FEV_1_ directly by altering the development of a normal-calibre airway, which would be expected to contribute to the risk of developing COPD. Alternatively, genetic variants associated with individuals who have already developed disease might affect the extent of tissue remodelling, such that more severe reductions in lung function parameters are seen in those carrying these variants. The available evidence suggests that the first of these explanations — altered lung function contributing to disease risk — may better explain the majority of available data, at least for COPD. Further support for this explanation comes from the observation that many of the genetic associations with lung function are present across age strata^8^ and that many implicate genes important in developmental pathways, suggesting that genetic control of normal lung development contributes to disease risk in later life^8,10,11,13,23^.

The observation that many individual genetic variants seem to contribute to more than one trait is probably explained by the molecular pathways often underlying multiple processes, such as lung development, lung inflammation and tissue remodelling. As such, identifying common pathways that are dysregulated in different traits may provide broader opportunities for therapeutic intervention.

## Gene–environment interaction in respiratory disease

Smoking is perhaps the most studied environmental risk factor in respiratory disease. Prior work has established that smoking has a causal role in COPD and is associated with the onset and exacerbation of asthma as well as with interstitial lung diseases, including IPF^74^. However, not all smokers develop COPD, strongly suggesting that smoking interacts with genetics to shape disease outcome. Indeed, a recent analysis of UK Biobank data showed that smoking exposure had a stronger effect on lung function amongst those with higher genetic risk (as measured by a PRS^75^). Importantly, a large number of well established genetic variants contribute to the risk of tobacco addiction (mostly pointing to central nervous system pathways or nicotinic receptor variants)^76^.

In addition to smoking, both indoor and outdoor air pollution are also major risk factors for respiratory diseases, suggesting that a better understanding of gene–air pollution interactions could have global public health implications. Studies attempting to investigate this topic have used both genome-wide and candidate-gene approaches to try to improve understanding of gene–environment interactions in respiratory disease (Table 2). A number of candidate gene studies have examined asthma–smoking and asthma–air pollution interactions, with a particular focus on antioxidants in the glutathione-*S*-transferase family^77^. However, the glutathione-*S*-transferase family has not been among the top findings from genome-wide interaction studies. Instead, such studies have identified evidence of gene–smoking interaction effects at known risk loci for lung function and COPD, such as *EGLN2* and *CHRNB4* (refs. 78,79). Studies using UK Biobank data have also identified gene–air pollution interactions with a clinically meaningful effect on lung function but these require replication in independent populations^80^. Finally, the largest GWAS of lung function identified 69 out of 1,020 genetic associations with nominal evidence of interaction with smoking. One of the strongest signals (at the *HTR4* locus) showed a 76% larger effect on one measure of lung function (FEV_1_) in people who had smoked compared with those who had not^22^.

These observations suggest that, although examples of gene–environment interactions do exist, genetic and environmental effects contributing to lung function can also be additive rather than synergistic (although larger sample sizes are needed to exclude small interaction effects). However, our understanding to date is limited, partly owing to the relatively small size of cohorts currently available, which leads to a lack of statistical power to detect effects. Researchers are also challenged by the need to disentangle the effects of the myriad combinations of potentially harmful compounds, degree of exposure, timing of exposure and other modifying factors. Overcoming this challenge may require approaches that extend beyond genetic associations between a single exposure and single disease trait to consider exposures holistically and incorporate other ’omics data^81^.

Mendelian randomization studies can also provide insight into the role of environmental risk factors in pathogenesis. Mendelian randomization can explore the use of genetic variants known to be associated with a given environmental exposure (for example, risk of addiction to tobacco smoking) as an ‘instrument’ with which to estimate the causal effect of that exposure on an outcome of interest^82^. In another example, genetic variants known to be associated with increased body mass index have been used to demonstrate that the observational association between body mass index and asthma is probably causal^83^. As the quantity of omics data increases, Mendelian randomization may also provide insight into the causal role of proteins in particular by utilizing genetic variants predictive of protein levels^84^. More specifically, Mendelian randomization approaches can potentially help to distinguish between whether altered protein levels contribute to the risk of developing a disease or whether altered protein levels are caused purely by the presence of disease. However, Mendelian randomization relies on key assumptions: in particular, Mendelian randomization assumes that the genetic variants influence the outcome only through the exposure instead of through alternative, pleiotropic effects. Careful selection of the genetic variants used for Mendelian randomization and testing of assumptions (for example, through sensitivity analyses) is therefore critical when using Mendelian randomization approaches.

## Applications for respiratory disease healthcare

Given the current state of knowledge, we next consider how genetic information might inform patient care.

### Preventative measures and early intervention

PRSs calculated from GWASs of respiratory diseases can be used to identify individuals or populations that could benefit from preventative measures or early intervention. However, although PRSs have reasonable predictive value, so far they have not generally been incorporated into guidelines or care pathways. One option worthy of more study is to combine PRSs with other known risk factors to improve prediction sensitivity and specificity. For example, a recent study found that combining PRSs with clinical risk factors (age, sex and history of smoking) improved prediction accuracy for COPD compared with a model that considered only clinical risk factors. This PRS was also associated with computed tomography image phenotypes, including percentage airway wall area and quantitative and qualitative measures of emphysema^85^. The potential power of this PRS to predict respiratory disease risk is illustrated by the observation that being in the top decile for the risk score for COPD is equivalent in absolute risk to a smoking history of approximately 20 cigarettes per day for 50 years. This PRS was not itself correlated with smoking history and therefore these effects are additive. However, given that we lack any disease-modifying interventions for COPD other than smoking cessation, it is unclear how PRSs could be used in current healthcare pathways.

### Personalized therapeutics

A clearer understanding of the genetics of respiratory disease could lead to improved therapeutic approaches for stratified or precision medicine. This concept relies on identifying subgroups of patients who are likely to respond better or worse to a given drug targeting a specific pathway. If a pathway is particularly relevant to disease in a subgroup of patients, as identified on the basis of their genetic profile, then therapies should theoretically be targeted to this subgroup. Such an approach could, for example, identify patients that respond better to a therapeutic agent because their genetic risk profile indicates that the relevant pathway is differentially regulated. Although this approach could reduce the market size for a new drug, the improved efficacy in a subgroup could still be beneficial overall, particularly if it meant that the drug were then to continue in development despite whole-population-based approaches showing limited efficacy. Notably, this approach is conceptually very similar to the existing strategy of stratifying asthma patients for anti-IL-5 therapy using the eosinophil count as a biomarker^86^. However, until now drug development for many targets in, for example, asthma — including, IL-5, IL-4 and TSLP — has not been driven mainly by the use of GWAS data, despite supporting genetic data being known to increase the likelihood of a drug reaching market^18^.

## Conclusions and perspective

The past 15 years have seen major advances in our understanding of the genetic basis of chronic respiratory disease. The challenge for the next decade is to uncover the mechanisms that drive the observed associations between genetic variants and the risk of developing respiratory disease or rate of disease progression.

Genetic information about a given disease provides valuable insights into disease mechanisms that are not built on preconceptions. Although genetic variants associated with respiratory disease risk have implicated many predictable genes on the basis of functional annotation, there are also many implicated genes that would probably not have been considered relevant. For example, one of the strongest genetic associations for both altered lung function and COPD is at the *NPNT* locus, which encodes nephronectin. Nephronectin is an important regulator of renal development and function in mice and humans^87^ but was not previously thought to have a role in the lung. This result has led to studies of nephronectin in lung development that have found that nephronectin may act through integrin α-8 to promote normal lobar development of the lung^88^. Thus, a better understanding of the genetic architecture of COPD will continue to provide insights into potential disease mechanisms.

Moving forward, a better understanding of respiratory disease genetics will be achieved through additional development in several areas. First, larger and more ethnically diverse cohorts will improve fine mapping and variant-to-gene mapping^29^. Second, increasing the number of respiratory-relevant exome and whole-genome sequencing datasets will enable rare variant analyses^28^. Third, collecting larger and more lung-specific ’omics datasets would be helpful. Examples include population-level RNA sequencing to measure expression quantitative trait loci as well as single-cell and spatial transcriptomics for higher resolution of expression profiles^89–91^. Additionally, proteomic data (see ref. 92) can help differentiate between variants that affect gene expression or post-translational regulation. Fourth, better integrating omic data with genetic association data should help inform variant-to-gene mapping. For example, as discussed above, Mendelian randomization techniques can be used to potentially address causality by defining SNP instruments associated with the expression level for proteins of interest^93^. Finally, better in vitro models of human lung development and repair will help to define the role of key genes more clearly in respiratory disease identified using the approaches described in this Review. Thus, by collecting more and better-quality genomics data, we will understand respiratory diseases better, which should lead to improved strategies for patient management.

## Figures and Tables

**Table 1:** Summary of heritability estimates and GWASs for key respiratory traits

Trait or disease	Approximate heritability estimates (%)	Number of GWASs^a^	Number of associations reported in all GWASs^a^	Largest GWAS sample to date	Ref.
FEV_1_	40–55%	57	2,298	580,869 participants	22
FEV_1_/FVC	55%	35	3,691	580,869 participants	22
FVC	40–45%	39	1,400	580,869 participants	22
COPD	30–40%	113	1,150	15,256 cases 47,936 controls	15
Asthma	60–70%	194	3,291	153,763 cases 1,647,022 controls	41
Asthma-COPD overlap	Not available	3^b^	8	8,068 cases 40,360 controls	53
IPF	30% (SNP-based)	42^c^	210^c^	11,160 cases 1,364,410 controls	66

a From the NHGRI–EBI GWAS Catalog (https://www.ebi.ac.uk/gwas/; accessed 22 December 2023).

b Asthma–COPD overlap is not described in the NHGRI–EBI GWAS Catalog so data were curated by authors^53,57,58^.

c The NHGRI–EBI GWAS Catalog combines interstitial lung diseases. COPD, chronic obstructive pulmonary disease; FEV_1_, forced expiratory volume in one second; FVC, forced vital capacity; IPF, idiopathic pulmonary fibrosis.

**Table 2:** Gene–environment effects from genome-wide interaction analyses in respiratory disease

Environmental stimulus	COPD or lung function	Asthma
Tobacco smoke	Significant interactions with GRS for COPD and lung function in the UK Biobank data; replication needed^75,94^.	Significant interaction at *KLH1,* with evidence of consistency across five large cohorts^96^.
	Significant, replicated interactions at known COPD risk loci *CHRNB4* and *EGLN2^78^.*	Several other loci with suggestive significance^7,97–99^.
	Significant but not replicated interactions at several other loci^78,95^.	
Outdoor air pollution	Significant but not replicated interactions between lung function and PM_2.5_, PM_10_ or NO_2_ at seven loci (such as *C1orf159, FAT1* and *KDM3B)^80^.*	Suggestive evidence of an interaction effect of NO_2_ on childhood asthma at *B4GALT5, ADCY2* and *DLG2^100^.*
	No interaction with a GRS for lung function^80^.	
Occupational dust and chemicals	Suggestive associations for lung function identified from cohorts that used self-reported job title as a proxy for exposure^101,102^.	No genome-wide gene-environment studies.

*P* < 0.05 or the Bonferroni-corrected equivalent was considered evidence of replication. COPD, chronic obstructive pulmonary disease; GRS, genetic risk score PM2.5; particulate matter under 2.5 μm; PM10, particulate matter under 10 μm.
